# Perivascular spaces balance vascular dimensions without impairing brain clearance mechanisms

**DOI:** 10.1186/s12987-026-00775-9

**Published:** 2026-02-14

**Authors:** Nina G. Smets, Bente van der Velden, Jenneke Oudijn, Lindy K. Alles, Gustav J. Strijkers, Erik N.T.P. Bakker

**Affiliations:** 1 Biomedical Engineering and Physics, Amsterdam UMC location University of Amsterdam, Meibergdreef 9, Amsterdam, The Netherlands; 2 Amsterdam Cardiovascular Sciences, Amsterdam, The Netherlands; 3 Amsterdam Neuroscience, Neurovascular Disorders, Amsterdam, The Netherlands; 4 Amsterdam Cardiovascular Sciences, Atherosclerosis and Aortic Disease, Amsterdam, The Netherlands; 5 Amsterdam Cardiovascular Sciences, Cardiomyopathy and Arrhythmia, Amsterdam, The Netherlands

**Keywords:** Vasodilatation, Cerebrospinal fluid, Perivascular spaces, Brain clearance, Hypercapnia, Microscopy

## Abstract

**Background:**

Perivascular spaces (PVS) contribute to brain clearance, as they connect the cerebrospinal fluid (CSF) in the subarachnoid space and ventricular system with the interstitial fluid. We tested the hypothesis that the size and exchange function of PVS depend on the degree of blood vessel dilatation.

**Methods:**

To induce vasodilatation, we subjected mice to hypercapnia and compared this to normocapnic conditions. The study comprised three experimental approaches. In the first series, cranial window surgery and two-photon microscopy were used to assess CO₂-induced changes in vascular and PVS morphology. The second series investigated tracer influx following cisterna magna tracer injection and 30-min exposure to either normocapnia or hypercapnia, with subsequent confocal imaging of brain slices. The third approach evaluated tracer efflux and parenchymal diffusion after intracortical injection via a cranial window port and widefield fluorescence imaging.

**Results:**

Arterial blood sampling confirmed hypercapnia. Hypercapnia led to vasodilatation in cerebral arteries, but not cerebral veins. The periarterial spaces balanced vasodilatation by a decrease in size. No effect was found on perivenous spaces. Despite changes in size of the PVS, no significant differences were found in CSF tracer influx from the subarachnoid space into the brain parenchyma, or subregions of the brain. Additionally, hypercapnia did not affect the efflux of fluorescent tracer from the cortex over the course of one hour.

**Discussion:**

Hypercapnia-induced vasodilatation was accommodated by shrinkage of the periarterial spaces. Veins and perivenous spaces were not affected. We propose that PVS might act primarily as a buffer for volume changes induced by vasomotor activity. Changes in PVS dimensions, induced by relatively mild vasomotor changes had no effect on brain clearance related parameters under the current experimental conditions.

**Supplementary Information:**

The online version contains supplementary material available at 10.1186/s12987-026-00775-9.

## Background

Impaired brain clearance has been associated with protein accumulation, which contributes to the development of neurodegenerative diseases including Alzheimer’s disease, Cerebral Amyloid Angiopathy, and Parkinson’s disease [1–3]. Perivascular spaces (PVS) have been hypothesized to contribute to the clearance of waste products such as amyloid-beta as they connect the cerebrospinal fluid (CSF) in the subarachnoid space (SAS) and ventricular system to the interstitial fluid (ISF) [1, 4]. Therefore, understanding the role of PVS in brain clearance is of great importance.

The exchange between CSF and ISF is dependent on several parameters, including brain state [5], arterial pulsations [6], vasomotion [7], and experimental conditions including anesthesia. Recent findings have shed light on the effects of different anesthetics on CSF flow and their influence on brain clearance [8, 9]. Specifically, isoflurane anesthesia has been observed to lower CSF influx compared to ketamine/xylazine anesthesia mixture [10]. Moreover, Ma et al. highlighted a clear difference in CSF flow under certain anesthetics compared to awake imaging [11]. Isoflurane is a known vasodilator, while dexmedetomidine and xylazine have been shown to exert vasoconstrictive effects through α2-agonist activity [12, 13]. These outcomes suggest that the observed changes in glymphatic parameters might be a consequence of the associated vasomotor changes [14, 15].

A link between vasodilatation and altered CSF–ISF exchange is further strengthened by experiments where mice were exposed to hypercapnia. Previous research by Goodman and Iliff reported a slower cervical lymphatic drainage of a fluorescent intraparenchymal tracer in hypercapnic mice [16]. Injection of the fluorescent tracer into the CSF reduced CSF–ISF exchange, but did not alter its drainage to the cervical lymphatics [16]. While the authors did not measure vascular diameter changes, these findings suggest a reduction in CSF–ISF exchange and lymphatic drainage as a result of vasomotor changes.

The present study tested the hypothesis that vasodilatation, achieved by inducing hypercapnia, reduces the dimensions of PVS and as a consequence, a reduction in overall CSF influx and efflux of tracer from the cortex. Mice were exposed to either a mixture of air and oxygen, or a mixture of air, oxygen, and CO_2_ to induce hypercapnia. Two-photon microscopy was used to study the effects of hypercapnia on the cerebral vasculature and the PVS, while whole-brain CSF tracer influx was analyzed using confocal imaging on brain slices. Finally, a tracer was injected into the cortex via a small access port in a cranial window to investigate the tracer distribution.

## Methods

### Animals

A total of 31 C57BL/6J mice of approximately 16 weeks of age were used. Animals were obtained from Envigo RMS (The Netherlands) and acclimated for at least seven days before the study. Animals, housed in groups with a 12 h light/dark cycle, had ad libitum access to food and water. This study was approved by the Academic Medical Center Animal Ethics Committee and was performed according to the ARRIVE guidelines and European Union guidelines for the welfare of laboratory animals (Directive 2010/63/EU). Animals were anesthetized before imaging with an induction of 4% isoflurane, after which the mice were injected intraperitoneally with a mixture of 125 mg/kg ketamine (Ketanest-S, Pfizer), 0.1 mg/kg dexmedetomidine (Dormitor, Orion Pharma), and 0.05 mg/kg atropine sulfate (Teva), diluted in 0.9% NaCl (KDA mixture). After the injection of the induction dose, isoflurane was gradually decreased to 0%. The induction dose was 0.0075 ml/g body weight, with a maintenance dose of 0.0025 ml/g body weight. To maintain appropriate physiological health, mice were mechanically ventilated using a tracheotomy before imaging. A mixture of 50% air and 50% oxygen was connected to the ventilator (RoVent, Kent Scientific). The breathing rate was set at 150 bpm and the tidal volume was set according to body weight (8 ml/kg). Mice were connected to an oxygen and heart rate monitor during the surgical procedure (MouseSTAT, Kent Scientific). Body temperature was regulated using a heating pad during all experiments. All mice were sacrificed using an overdose of three times the KDA mixture induction dose.

### Experimental design

The present study consists of three separate experimental approaches, each using the two different conditions. Mice were subjected to either a normocapnic state, defined as a mixture of 50% air and 50% oxygen, or a hypercapnic state, characterized by a mixture of 47.5% air, 47.5% oxygen, and 5% CO_2_. The first group of mice underwent cranial window surgery. Two-photon microscopy was used to determine the effects of CO_2_ on blood vessel and PVS morphology (*n* = 5; 2 male, 3 female). In order to measure the effects of vasodilatation on the influx of CSF tracer, a separate set of animals underwent fluorescent CSF tracer injection through the cisterna magna (*n* = 12; 6 male, 6 female). Subsequently, the animals were administered either the normocapnic or the hypercapnic mixture for a duration of 30 min. Arterial blood samples were measured to confirm the hypercapnic state, and the animals were fixed for confocal imaging. Finally, the third objective was to determine the effect of vasodilatation on the efflux of tracer as a measurement of brain clearance (*n* = 14; 7 male, 7 female). A cranial window with a silicone port enabled intracortical injection of a fluorescent tracer. Tracer movement and outflow were recorded with a widefield fluorescence camera, while mice were exposed to either normocapnic or hypercapnic gas mixtures.

### Cranial window surgery

One day before the cranial window surgery until three days after surgery, 0.06 mg/ml of carprofen (Rimadyl) was added to the drinking water. The surgery was performed under 2% isoflurane with 70% air and 30% oxygen. Mice were then fixated in a stereotactic frame and received a subcutaneous injection of 0.01 ml/g body weight carprofen (0.5 mg/ml, Rimadyl). A subcutaneous injection of 1.5 mg/kg ropivacaine (0.5 mg/ml, Fresenius Kabi) was given under the skin on the skull to provide additional local anesthesia. Skin and the periosteum were then removed from the skull. A circle of 3 mm diameter was made above the right middle cerebral artery (MCA) using a dental drill with a round tip. During the drilling process, the skull was repeatedly cooled with PBS. The circular cranial bone window was then removed carefully and replaced with a 3-mm-diameter cover glass. For the experiments involving intracortical injection, a cover glass with a silicone-filled port (Kwik-Sil) was used, as previously described by Roome and Kuhn [17]. After surgery, mice again received a subcutaneous injection of 0.01 ml/g body weight carprofen (0.5 mg/ml, Rimadyl) and had a recovery period of at least 7 days.

### Intracisternal tracer injection

Following induction with the KDA mixture and initiation of mechanical ventilation, 1% xylocaine was applied to the neck for local anesthesia, after which the neck muscles were separated to expose the cisterna magna. 15 µl of FITC-dextran 70 kDa (5 mg/ml in aCSF, Invitrogen, fixable, D1822) was injected with a 30-gauge needle into the CSF at a rate of 1 µl/min using an infusion pump (Harvard Apparatus). The needle in the cisterna magna was then secured with tissue glue to prevent movement. To visualize blood vessels, 100 µl of Texas Red-dextran 70 kDa (10 mg/ml in PBS, Invitrogen, fixable, D1864) was injected into the bloodstream via a retro-orbital injection.

### In vivo two-photon microscopy

We used a Leica multiphoton microscopy system (Leica Microsystems, Wetzlar, Germany) with an adjustable Insight DeepSee (SpectraPhysics) laser for imaging the blood vessels and CSF tracer in the brain. Images were obtained using a 25x water immersion objective (NA = 0.95). FITC-dextran was excited at 790 nm wavelength, whereas Texas Red-dextran was excited at 910 nm. Two HyD detectors captured the emitted light using a FITC-TRITC filter cube with a beam splitter at 560 nm. Laser power was increased accordingly for imaging deeper into the brain parenchyma. Z-stacks were acquired at 1024 × 1024 pixel resolution with a step size of 2 μm. Imaging was performed between 15 min and 1 h after end of infusion of the tracer into CSF. Similar z-stacks were acquired under two conditions, using either a 50% air / 50% O₂ mixture or a 47.5% air / 47.5% O₂ / 5% CO₂ mixture, in randomized order. Image analysis was performed using Fiji ImageJ software [18]. Arteries were distinguished from veins based on the morphology, thickness of the vessel wall, and location. Vessels with unclear classification were excluded. Each vascular bifurcation was treated as a separate segment and counted as a new data point. Image stacks were rotated to obtain 90° cross-sections, and binary images were generated. These images were denoised, after which blood vessel and perivascular areas were segmented manually in a blinded manner. An example of the segmentation can be found in Supplementary Fig. 1.

### Arterial blood sample and perfusion fixation

For confocal imaging, mice were exposed for 30 min to either the control gas mixture or the CO₂-enriched mixture, including the tracer infusion period. The mice received an intraperitoneal injection of 100 µl of 4% heparin, 5 min before killing. After the 30 min period, the thorax was opened and an arterial blood sample was taken from the left ventricle. The arterial blood gas values were measured immediately (RAPIDPoint 500e, Siemens Healthineers). The mice received an overdose of 3 times the induction dose of the KDA mixture, and a perfusion needle was immediately inserted in the left ventricle of the heart to start the perfusion fixation. The right atrium was punctured for outflow. Mice were perfused with 15 ml of PBS, and then 15 ml of 4% paraformaldehyde (PFA). The brain was extracted carefully for further processing. Brain tissue was kept in 4% PFA for 24 h at 4 °C. Then, brains were transferred to a 30% sucrose solution for at least 48 h at 4 °C to ensure cryoprotection. The midbrain was stored at -80 °C until further processing.

### Brain sectioning and confocal microscopy

Brain slices of 50 μm thickness were obtained using a cryostat (Thermo Scientific CryoStar NX70). Brain slices were put in a cryoprotectant solution. For analysis, similar brain slices were selected from each brain, washed with PBS, and stained with bisbenzimide diluted in PBS (1:100, Hoechst 33342, Invitrogen, 3.5 mg/ml). Brain sections were placed on permafrost microscopy slides with mounting medium (S3023, Dako).

A Leica Stellaris confocal microscope with a 20x/0.75 dry objective was used to image the brain slices. The cell nuclei were visualized using a 405 nm excitation wavelength, and the FITC-dextran was imaged using a 495 nm wavelength. Settings including laser power and gain were kept constant across all brain sections. Z-stacks of the whole brain were made using step size of 2 μm and a resolution of 256 × 256 pixels. Fiji ImageJ software was used to analyze the images [18]. Brain regions were determined using the Allen Brain Atlas [19]. The following brain regions were considered: brain surface, total brain parenchyma (brain parenchyma without SAS), cerebral cortex, cerebral nuclei (striatum and pallidum), hypothalamus, and thalamus. The ratio of the integrated density of the brain parenchyma to the integrated density of the SAS was calculated, as well as the ratio of integrated density of the subregions compared to the SAS.

### Intracortical tracer injection

Mice undergoing widefield fluorescent camera imaging received an intraparenchymal injection of 1 µl of FITC-dextran 70 kDa (5 mg/ml in artificial CSF, Invitrogen, fixable, D1822). The Hamilton 34-Gauge needle was then placed above the silicone port in the cranial window, and the injection was performed at a depth of 1 mm into the cerebral cortex. The fluorescent tracer was injected into the cortex at a rate of 0.1 µl/min using an infusion pump (Harvard Apparatus). After the infusion process was completed, the needle was carefully extracted, and the animal was then positioned under the widefield fluorescent camera.

### Widefield fluorescent imaging

Following the intracortical injection, mice were placed under a microscope connected to a high-speed camera (ORCA-Flash4.0 C11440-22CU, Hamamatsu Photonics, Japan) with a 10x (NA = 0.3) dry objective. The experimental setup consisted of a filter cube featuring an excitation spectrum ranging from 450 to 490 nm, with an emission detection window set at 515 nm. Images were captured at a pixel resolution of 2048 × 2048 and an exposure time of 10 ms. All settings were kept constant across all mice. A baseline image was created, and subsequently, an additional image was captured at 10-min intervals for a duration of one hour. The amount of bleaching due to image capturing was tested beforehand and was found negligible. Following this, the mice were killed using an overdose of the KDA mixture and perfusion fixed, as described above.

The analysis of the acquired images was conducted using Fiji ImageJ software [18]. The injection site and the silicone area were excluded from the images. Any air bubbles and other disruptions were also excluded. Integrated density was expressed as a percentage relative to baseline (t = 0, set at 100%) and compared across regions and time points. The window was divided into the following four regions: (1) The total area of the window, excluding the silicone area (2). Zone 1 corresponds to the inner ring surrounding the silicone area (3), zone 2 is defined as the middle ring, and (4) zone 3 is the outer ring (see Fig. 2d). For each brain, the integrated density of a small region in the darkest part of the window was measured as background signal and subtracted from all values.

### Statistical analysis

All statistics were performed using IBM SPSS Statistics 28 and Graphpad Prism 10 software. Data was checked for normal distribution using the Shapiro-Wilk test. Segmented blood vessel and PVS data were rank-transformed and analyzed using a linear mixed model with compound symmetry. The ratios of SAS and different brain regions were tested using a one-way ANOVA. The percentage change in fluorescence level regarding the in vivo tracer efflux was analyzed using repeated measures ANOVA with the Greenhouse-Geisser correction. All values are presented as mean ± standard deviation with statistical significance defined as *p* ≤ 0.05.

## Results

### Vasodilatation decreases the size of periarterial spaces

**Fig. 1:**
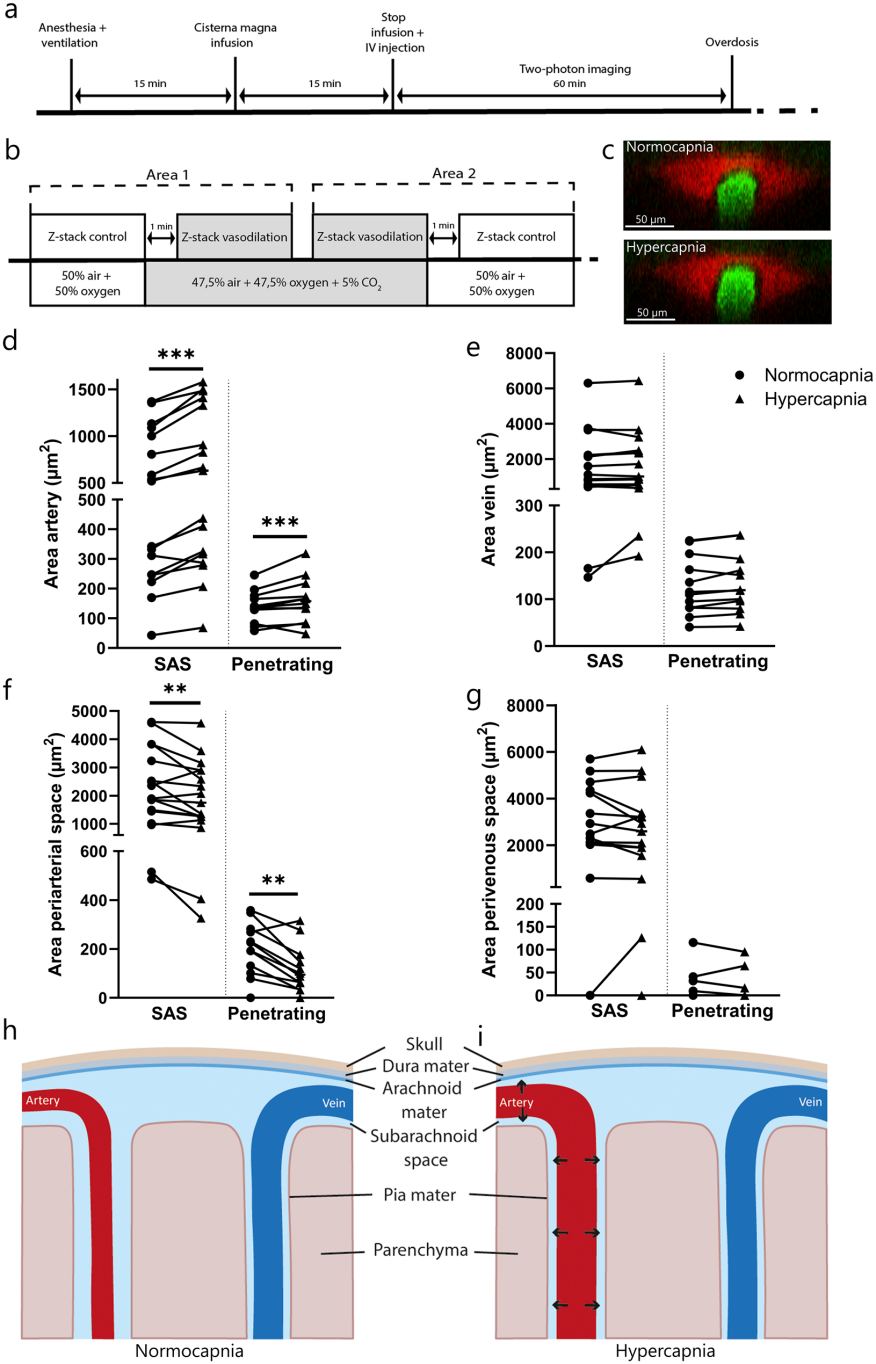
The effect of hypercapnia on blood vessels and perivascular spaces using two-photon microscopy. (**a**) Overview of experimental design. (**b**) Experimental two-photon microscopy setup with randomized conditions of normocapnia and hypercapnia (*n* = 5). (**c**) Example cross-sectioned two-photon image of an artery with perivascular space under normocapnic and hypercapnic conditions. (**d**) The area of the artery (*n* = 17) and (**e**) veins (*n* = 15) under normocapnia and hypercapnia for blood vessels in the subarachnoid space (SAS) and penetrating vessels. (**f**) The area of the periarterial spaces (*n* = 12) and (**g**) perivenous spaces (*n* = 12) under normocapnia and hypercapnia for perivascular spaces in the SAS and penetrating vessels. Data is only separated by location for visual purposes. Schematic representation of the observed differences in vessel and PVS sizes between (**h**) normocapnic and (**i**) hypercapnic conditions. *p ≤ 0.05, **p ≤ 0.01, ***p ≤ 0.001

To visualize the effects of vasodilatation on PVS dimensions, two-photon microscopy was conducted under normocapnic and hypercapnic conditions in the same animal (Fig. 1a and b). A total of 56 blood vessels with their corresponding PVS were segmented, including 17 arteries and 15 veins in the SAS, and 12 arteries and 12 veins penetrating the brain tissue. Arteries demonstrated significant enlargement in response to hypercapnia, while veins exhibited no substantial change in cross-sectional area (Fig. 1d and e). This finding confirmed the effectiveness of the intervention in inducing vasodilatation. This change was independent of the location of the blood vessel; blood vessels in the SAS exhibited a comparable response to the stimulus as blood vessels that penetrated the brain tissue. As a result, the dataset was not divided based on blood vessel location for further statistical analysis. This subdivision was applied only in the figures, to account for differences in vessel size and to aid visualization. Interestingly, the area of periarterial spaces decreased in response to vasodilatation, while the perivenous spaces remained constant (Fig. 1f and g). Notably, in 8 out of 12 veins, no perivenous space was present. Figure 1h and i schematically summarize the observed differences in vessel and PVS sizes between normocapnic and hypercapnic conditions. The percentual changes in blood vessel and perivascular space area due to hypercapnia are provided in Supplementary Fig. 2.

### Vasodilatation has no effect on tracer influx into the brain

**Fig. 2:**
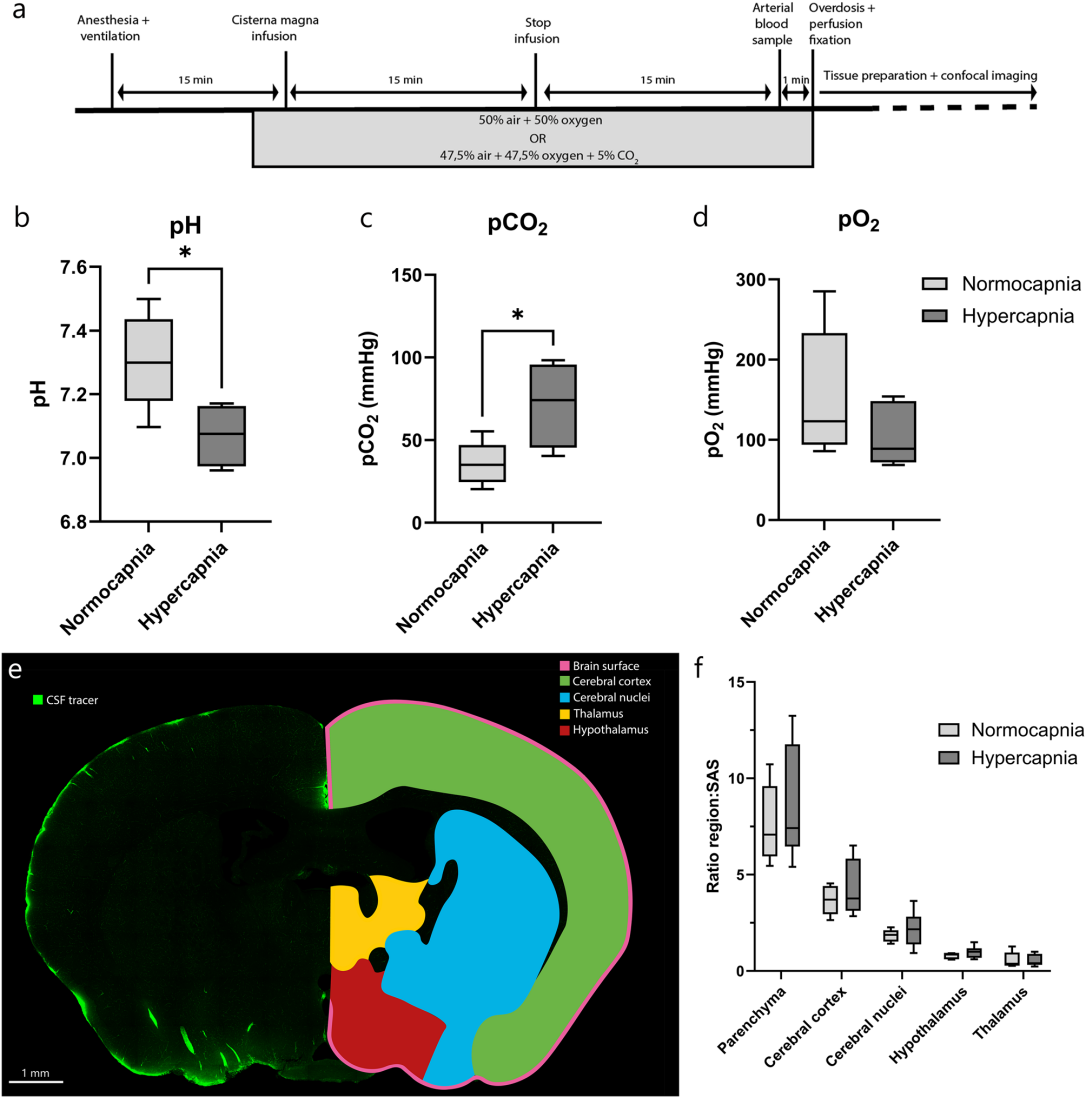
CSF-ISF exchange after 30 min between normocapnic and hypercapnic mice. (**a**) Experimental design. (**b**) pH, (**c**) pCO_2_, and (**d**) pO_2_ levels after 30 min in normocapnic (*n* = 5) and hypercapnic mice (*n* = 4) as measured by arterial blood gas samples. (**e**) Confocal image of an example brain slice with CSF tracer (green) and the segmentation regions on the right. (**f**) The ratio of integrated density between the different brain regions and the Subarachnoid Space (SAS) for both normocapnic (*n* = 5) and hypercapnic mice (*n* = 6). b-d: Analyzed using unpaired t-test, f: one-way ANOVA. *p ≤ 0.05, **p ≤ 0.01, ***p ≤ 0.001

To determine whether vasodilatation would affect tracer influx, mice received an intracisternal injection and were exposed to either the normocapnic or hypercapnic gas mixture for a period of 30 min. Afterwards, arterial blood gas samples were collected and the brains were analyzed using confocal microscopy. The pH levels demonstrated a substantial decrease in hypercapnic mice (7.306 ± 0.15 in normotensive mice vs. 7.071 ± 0.10 in hypercapnic mice; Fig. 2b), accompanied by a significant increase in their pCO_2_ levels (35.62 ± 13.02 mmHg vs. 71.75 ± 26.25 mmHg in normocapnic and hypercapnic mice, respectively; Fig. 2c). The pO_2_ levels were not different for both interventions (Fig. 2d). These findings validated the effectiveness of the gas challenge in yielding the expected outcomes.

Analysis of the confocal images of the brain was conducted by calculating the ratio of tracer signal in several brain regions to the signal present in the SAS. An overview of all brain regions can be found in Fig. 2e. No significant differences were found in CSF tracer level of the brain parenchyma between the normocapnic and hypercapnic mice. Furthermore, the cerebral cortex, cerebral nuclei, hypothalamus and thalamus exhibited no significant change in CSF tracer levels due to hypercapnia (see Fig. 2f).

### No change in tracer efflux due to vasodilatation

**Fig. 3:**
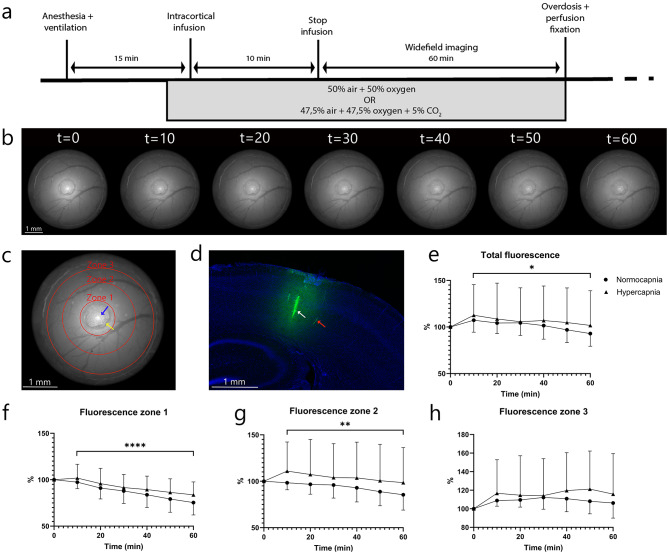
In vivo efflux of intracortically injected fluorescent tracer using widefield imaging. (**a**) Experimental design. (**b**) Example of the injected fluorescent tracer in the cranial window over time. (**c**) An example of the different zones used for further analysis. The blue arrow indicates the injection spot and the yellow arrow points to the silicone port. (**d**) Confocal image of a brain slice showing the injected tracer after one hour. The white arrow indicates the injection site and the red arrow points to the accumulation of fluorescent tracer around penetrating blood vessels. (**e**) Percentage decrease of fluorescence for normocapnic (*n* = 7) and hypercapnic mice (*n* = 7) in the total cranial window, (**f**) zone 1, (**g**) zone 2, and (**h**) zone 3. *p ≤ 0.05, **p ≤ 0.01, ***p ≤ 0.001, ****p ≤ 0.0001

Whether vasodilatation alters tracer efflux pathways was investigated using an intracortical injection via a small silicone port in the window, and in vivo tracer efflux was visualized over one hour under either normocapnic or hypercapnic conditions. Figure 3b shows an example of tracer distribution over the course of one hour. To determine whether the tracer signal was cleared or dispersed over the brain tissue, the windows were separated into three zones (Fig. 3c). Interestingly, post-mortem analysis of the injection site revealed a diffuse cloud of fluorescent tracer within the brain tissue, along with clearly labeled blood vessels in the region (Fig. 3d). Total fluorescence in the window decreased over time but did not differ between normocapnia and hypercapnia (Fig. 3e). Furthermore, fluorescence levels in zones 1 and 2 also decreased significantly over time. No significant differences across the three zones were observed between normocapnic and hypercapnic mice (Fig. 3f-h). The calculated travel distance of FITC–dextran, based on its diffusion coefficient [20] was consistent with the distance observed in the cranial window. This suggests that tracer efflux in this study was governed primarily by diffusion rather than bulk flow.

## Discussion

### Periarterial spaces decrease in size upon vasodilatation

The objective of this study was to establish the impact of vasodilatation on PVS dimensions and brain clearance-related parameters. Cerebral blood vessels and their corresponding PVS were segmented under normocapnic and hypercapnic conditions using two-photon microscopy. A strength of this study is that mice served as their own controls, with the same vessels measured under both normocapnia and hypercapnia applied in random order. Arteries responded to the CO₂ challenge with vasodilatation, and the observed diameter changes were comparable to those reported by others [21, 22]. No such effect was observed in cerebral veins, which was expected since perfusion is primarily regulated on the arterial side of the circulation [23]. Arterial vasodilatation was accompanied by a reduction in the size of adjacent periarterial spaces, seemingly consistent with a compensation for the increased arterial diameter. This phenomenon was observed in both pial and penetrating arteries. These observations are in agreement with work by Bojarskaite et al., who showed similar compensatory changes in response to sleep cycle-associated changes in vessel diameter [24]. As the findings of our study reflect a static condition, it would be of interest to determine whether these observations also apply to dynamic conditions of vasoconstriction and dilation, including vasomotion. Furthermore, the impact of anesthesia warrants consideration, and a comparison of our results with data obtained in the unanesthetized state is a worthwhile direction for further research. We also found that cerebral veins did not exhibit such a response to the CO₂ challenge, and perivenous spaces remained unchanged accordingly. Moreover, we noted that perivenous spaces were often absent, especially around penetrating veins [25].

### Hypercapnia does not affect tracer influx

Given that hypercapnia affected PVS dimensions, we next studied the effects of hypercapnia on tracer influx into the brain parenchyma. After tracer infusion into the cisterna magna, no differences in tracer influx into the brain parenchyma or specific subregions were observed between normocapnic and hypercapnic groups. These results appear to contrast with previous work, which reported higher CSF influx in normocapnic than in hypercapnic mice using a similar approach [16]. That study suggested that vasodilatation induced by hypercapnia may limit CSF-ISF exchange. The discrepancy with our findings remains uncertain but may relate to methodological differences. The most important of these is that in the study of Goodman and Iliff, the animals were ventilated with 100% oxygen or 95% oxygen and 5% CO₂ to induce hypercapnia. In our study, we used a mixture of 50% oxygen and 50% air. The impact of this difference is clearly reflected in the arterial pO_2_ levels. These were around 440 mmHg in the paper of Goodman, while in our study, this value is around 100 mmHg, which is similar to physiological values. The response of cerebral blood vessels to hyperoxia is vasoconstriction [26]. Thus, the impact of hypercapnia in the study of Goodman may have been larger compared to our study, going from a more constricted state induced by hyperoxia to hypercapnia-induced dilation.

The lack of effect of hypercapnia on brain tracer influx is consistent with a SPECT study in rats, which found no significant differences in intracranial tracer distribution between normal ventilation and hypercapnic conditions [27]. That study also used a longer imaging time, thereby indicating that an extension in the imaging time would not result in a significant alteration in our results. Those authors argued that vasomotor effects influence clearance parameters but suggested that anesthesia may blunt the impact of hypercapnia. Overall, the effect of hypercapnia on brain tracer influx appears inconsistent across studies.

### Hypercapnia does not affect tracer efflux in vivo

In order to assess the impact of hypercapnia on tracer efflux, a fluorescent tracer was injected into the cerebral cortex of normocapnic or hypercapnic mice and then observed for a period of one hour. There was an initial rise in tracer signal, which we speculate was the result of tracer emerging at the brain surface from the deeper injection site. After that, we found a significant decrease in tracer level in both groups over time. However, no significant difference was found between the normocapnic and hypercapnic mice, indicating that vasodilatation did not affect the efflux of tracer from the brain tissue. We found a relatively slight decrease in the total fluorescence signal in the field of view. This suggests little efflux from the tissue. We speculate that the decrease in total fluorescence results from tracer reaching the CSF, from where it may be swept away. We did note tracer dispersion in the field of view, which was consistent with diffusion through the brain parenchyma, rather than bulk flow. Thus, PVS dimensions thus did not affect the efflux of tracer from brain tissue under the present study design. It should be noted that the current study used a static vasodilatation, whereas a more dynamic intervention protocol to induce vasomotor responses could affect the tracer efflux differently. For instance, an increase in the number and amplitude of vasomotion waves was previously shown to increase both the influx of CSF as well as tracer clearance rates [7, 28]. Despite the tracer being dispersed over time, this tracer distribution rate was much lower than that of the extravasated tracer in an earlier study, possibly due to a difference in tracer size [7]. In post-mortem brain sections obtained during the current study, we found that the tracer had spread into the brain parenchyma after injection in the cortex. Interestingly, PVS surrounding the cerebral vessels in the area became apparent with tracer. This could be the result of partial volume effects, with extracellular fluid accounting for most of the perivascular space volume, in contrast to tissue extracellular space taking up only a fraction of the volume. Alternatively, the strong perivascular signal could reflecting its role in facilitating clearance. However, given the static nature of the image, it is not possible to draw firm conclusions from these data regarding PVS as influx of efflux pathways.

### A role for the PVS beyond brain clearance?

We observed a consistent inverse relationship between changes in blood vessel diameter and size of the PVS. At the level of the whole brain, the Monro-Kellie doctrine states that intracranial volume remains constant due to rigidity of the skull. Thus, an increase in volume of either brain tissue, cerebrospinal fluid, or blood must be compensated by a decrease in volume of one of the other compartments [16]. In support of this, earlier research demonstrated a decrease in CSF volume in hypercapnic humans [29]. Moreover, an increased distribution of fluorescent tracer to the thoracic spine during hypercapnia was found in rats, possibly due to shrinking of the above-mentioned periarterial spaces [27]. We speculate that it is this principle of maintenance of volume that also acts on a local scale between arteries and their adjacent PVS. Local vascular dilatation may be facilitated by adjacent PVS that can readily accommodate a volume decrease, whereas vasoconstriction may be aided by CSF influx into the PVS. This mechanism could also explain why PVS are typically larger around arteries than veins, and why penetrating veins often lack a PVS [25]. After all, regulation of blood flow is a primary role of arteries, not veins, and therefore a PVS may be needed around arteries only for this purpose.

### Limitations

Hypercapnia may induce effects other than vasodilatation, such as acidification. However, the impact of a decrease in pH on clearance parameters is currently unknown. Second, the injected tracer volume is relatively high compared to the total CSF volume. However, in accordance with other work [30], our preliminary experiments indicated that a lower volume often failed to reach the dorsal surface of the brain in several mice, making this volume necessary. To minimize any influence of the infusion itself, all measurements were performed at least 15 min after tracer administration. Post-mortem analysis of CSF tracer can also be confounded by initial collapse of arteries and subsequent collapse of PVS [11, 25, 31]. To minimize this potential artifact, perfusion fixation was initiated immediately after administration of the overdose. Moreover, because the same protocol was applied to both groups, any such post-mortem effects are expected to be comparable across groups and minimally affect signal in the parenchyma [11]. In addition, heart rate and blood pressure were not measured, although both may be affected by hypercapnia and could have influenced tracer distribution. Finally, the type and depth of anesthesia strongly influence CSF flow and tracer diffusion in the brain. Both isoflurane and ketamine/xylazine have been shown to reduce parenchymal tracer penetration compared with the awake state [32]. Nonetheless, ketamine/xylazine, similar to the ketamine/dexmedetomidine regimen used in our study, has been shown to promote greater contrast penetration into the brain parenchyma and enhance CSF flow compared with isoflurane [10]. Even though isoflurane is a vasodilator and results in a diminished CSF flow [15, 32], our study suggests that its impact is not just a result of a change in dimensions of PVS. Other effects of isoflurane, such as an inhibition of vasomotion, could also alter tracer dispersion. Future research should focus on the effects of hypercapnia in an awake-state to completely rule out all effects of anesthesia on vasomotor activity and CSF flow.

## Conclusion

This study showed that vasodilatation reduced the size of periarterial spaces but did not affect tracer influx or efflux under static conditions, indicating that PVS size alone does not determine brain clearance pathways. We did find a strong inverse correlation between vasomotor changes and PVS size, suggesting an important role for PVS in local volume regulation. This relationship was observed in arteries, not veins, consistent with the central role of arteries in regulating blood flow.

## Supplementary Information

Below is the link to the electronic supplementary material.

Supplementary Material 1

## Data Availability

The datasets used and/or analyzed during the current study are available from the corresponding author on reasonable request.

## Abbreviations

CSF: Cerebrospinal fluid
ISF: Interstitial fluid
MCA: Middle cerebral artery
PFA: Paraformaldehyde
PVS: Perivascular space
SAS: Subarachnoid space
