# Supplementary material for: Perivascular spaces balance vascular dimensions without impairing brain clearance mechanisms
Source: Fluids Barriers CNS. 2026 Feb 14;23:33. doi: 10.1186/s12987-026-00775-9 (PMC12927245; doi:10.1186/s12987-026-00775-9)
Supplement: Supplementary file 1 — Supplementary Material 1 [file 12987_2026_775_MOESM1_ESM.docx]

**Supplementary Figures Manuscript ‘Perivascular Spaces Balance Vascular Dimensions Without Impairing Brain Clearance Mechanisms’**

Supplementary Figure 1


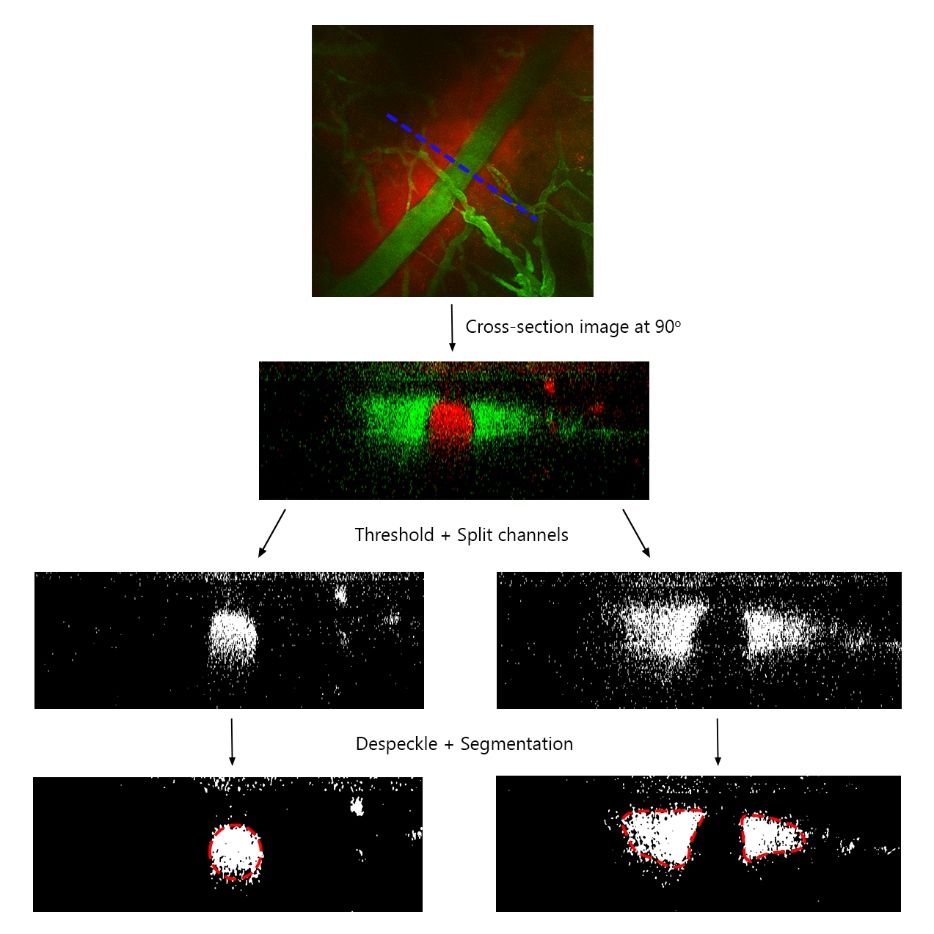


Supplementary Figure 1. Example of the segmentation pipeline of the two-photon microscopy images. The blue dashed line represents the cross-section obtained at a 90-degree angle from the blood vessel. The channels were divided so that both fluorescent tracers were depicted separately. Subsequently, the images were thresholded at equivalent levels to create a binary image. Afterwards, images were despeckled and segmented (red dashed line) in order to calculate the area. Image analysis was performed using Fiji ImageJ software.

Supplementary Figure 2


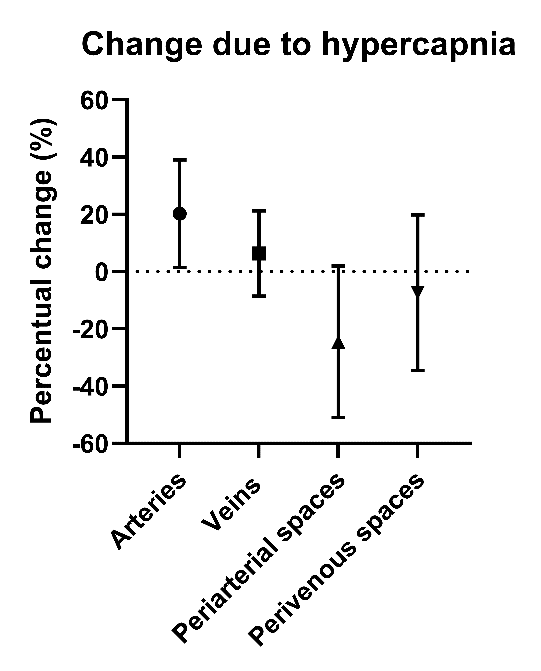


Supplementary Figure 2. Percentual change in blood vessel and perivascular space area between normocapnia and hypercapnia as measured by two-photon microscopy. Arteries (n=29), veins (n=27), periarterial spaces (n=29), perivenous spaces (n=27), obtained from five mice.
